# The Prognostic Value of Natural Killer Cells and Their Receptors/Ligands in Hepatocellular Carcinoma: A Systematic Review and Meta-Analysis

**DOI:** 10.3389/fimmu.2022.872353

**Published:** 2022-04-07

**Authors:** Jun-Shuai Xue, Zi-Niu Ding, Guang-Xiao Meng, Lun-Jie Yan, Hui Liu, Hai-Chao Li, Sheng-Yu Yao, Bao-Wen Tian, Zhao-Ru Dong, Zhi-Qiang Chen, Jian-Guo Hong, Dong-Xu Wang, Tao Li

**Affiliations:** ^1^ Department of General Surgery, Qilu Hospital, Shandong University, Jinan, China; ^2^ Department of Hepatobiliary Surgery, The Second Hospital of Shandong University, Jinan, China

**Keywords:** natural killer cells, receptor, ligand, hepatocellular carcinoma, prognosis

## Abstract

**Background:**

Natural killer (NK) cells play major roles in eliminating tumor cells. Preliminary studies have shown that NK cells and their receptors/ligands have prognostic value in malignant tumors. However, the relevance of NK cells and their receptors/ligands level to the prognosis of hepatocellular carcinoma (HCC) remains unclear.

**Methods:**

Several electronic databases were searched from database inception to November 8, 2021. Random effects were introduced to this meta-analysis. The relevance of NK cells and their receptors/ligands level to the prognosis of HCC was evaluated using hazard ratios (HRs) with 95% confidence interval (95%CI).

**Results:**

26 studies were included in the analysis. The pooled results showed that high NK cells levels were associated with better overall survival (HR=0.70, 95%CI 0.57–0.86, P=0.001) and disease-free survival (HR=0.61, 95%CI 0.40-0.93, P=0.022) of HCC patients. In subgroup analysis for overall survival, CD57^+^ NK cells (HR=0.70, 95%CI 0.55-0.89, P=0.004) had better prognostic value over CD56^+^ NK cells (HR=0.69, 95%CI 0.38-1.25, P=0.224), and intratumor NK cells had better prognostic value (HR=0.71, 95%CI 0.55-0.90, P=0.005) over peripheral NK cells (HR=0.66, 95%CI 0.41-1.06, P=0.088). In addition, high level of NK cell inhibitory receptors predicted increased recurrence of HCC, while the prognostic role of NK cell activating receptors remained unclear.

**Conclusion:**

NK cells and their inhibitory receptors have prognostic value for HCC. The prognostic role of NK cell activating receptors is unclear and more high-quality prospective studies are essential to evaluate the prognostic value of NK cells and their receptors/ligands for HCC.

## Introduction

Hepatocellular carcinoma (HCC) is the sixth most common malignancy worldwide and the third leading cause of cancer-related mortality (1). The major risk factors for HCC involve chronic hepatitis B and hepatitis C infection, alcohol, and metabolic liver disease (2). Natural killer (NK) cells, characterized as CD3^-^CD56^+^ lymphocytes, are mainly involved in the early defense against virus infections and play major roles in eliminating tumor cells (3). NK cells account for only about 5–20% of the circulating lymphocytes in the peripheral blood. In contrast, NK cells are abundant in human liver, accounting for almost half of intrahepatic lymphocytes (4), which lays foundation for the powerful role of NK cells in the liver tumor microenvironment.

Human NK cells are divided into two major subpopulations based on the surface density of CD56 antigen (5). CD56^dim^ NK cells display a mature phenotype, accounting for approximately 90% of all NK cells and mediating the cytolytic response, while immature CD56^bright^ NK cells account for 5%-15% of total NK cells and are regarded as cytokine producers (6). Another surface marker is CD57, which is a marker for differentiated and highly cytotoxic NK cells, and is described as a phenotypically stable NK cells marker (7).

The regulation of NK cell function is mediated by a series of activated or inhibitory surface receptors. The major activated receptors involved in target cell killing are NK group 2 member D (NKG2D) and natural cytotoxic receptors (NCRs). NCRs mainly consist of NKp44, NKp46 and NKp30 (8), and can recognize ligands from different sources, including viral, parasitic, bacterial, as well as cellular ligands, such as HLA-B-associated transcript 3/Bcl-2-associated athanogene 6 (BAT3/BAG6), mixed lineage leukemia 5 (MLL5), proliferating cell nuclear antigen (PCNA) and B7 homolog 6 (B7-H6) (9, 10). In contrast, NKG2D mainly binds to the major histocompatibility complex class I chain-related protein A and B (MICA and MICB) and UL16-binding proteins (ULBPs). After binding, it can activate NK cells to produce cytotoxic substances to kill harmful and tumor cells (11). Other activated receptors include CD16, NKp88, CD244, CD226 and cytokine receptors such as interleukin (IL)-2R, IL-12R, IL-28R, IL-18R, IL-1R8, IL-15R, IL-10R, interferon receptor (IFNR) and tumor growth factor-β receptor (TGF-βR) (12–14).

The major inhibitory receptors involved in target cell killing are NKG2A, CD96, killer immunoglobulin-like receptors (KIRs), T cell immunoglobulin and immunoreceptor tyrosine-based inhibitory motif domain (TIGIT) and T cell immunoglobulin domain and mucin domain-3 (TIM-3) (12). Other inhibitory receptors include programmed cell death-1 (PD-1), lymphocyte activation gene-3 (LAG3), leukocyte-associated immunoglobulin-like receptors (LAIRs), adenosine 2A receptor (A2AR) and immunoglobulin-like transcripts (ILTs) (15). PD-1 is primarily expressed by activated T lymphocytes, but may also be expressed by NK cells in tumor patients. PD-1/Programmed cell death ligand-1 (PD-L1) interactions can inactivate T cells and NK cells, allowing tumor cells to escape immune surveillance (16). Human histocompatibility leucocyte antigen E (HLA-E) is the main ligand of NKG2A, and is generally upregulated in cancer patients and predicts poor prognosis (17–19). The major histocompatibility complex class I (MHC-I) is the main ligand of KIRs, and is expressed on healthy hepatocytes. It interacts with inhibitory receptors on NK cells to prevent the activation of NK cells (14).

Until now, the correlation of NK cells and their receptors/ligands with the prognosis of HCC remains controversial (20–28). The purpose of this meta-analysis and review is to evaluate the prognostic value of NK cells and their receptors/ligands in HCC.

## Materials and Methods

### Search Strategy and Study Selection Criteria

This meta-analysis was conducted according to the Preferred Reporting Items for Systematic reviews and Meta-Analyses (PRISMA) guidelines (supplementary PRISMA Checklist) (29), and inclusion criteria were based on the PICOS model.

Relevant studies were independently searched by two authors (JSX, ZND) from the PubMed, Embase, Web of Science and Cochrane Library literature databases from the beginning of the database until November 8, 2021. Detailed search strategy was as described in the supplement. Additional articles were identified by a manual search of the references of eligible articles.

Studies were included if they met the following criteria. (1) all patients were identified as having HCC; (2) studies revealed the expression of NK cells and their receptors/ligands and obtained their levels by assaying; (3) studies provided adequate information to evaluate the hazard ratio (HR) and 95% confidence interval (95% CI); (4) the prognostic indexes such as overall survival (OS), cancer-specific survival (CSS), disease-free survival (DFS), recurrence-free survival (RFS), time-to recurrence (TTR), and progression-free survival (PFS) were evaluated; (5) Anti-tumor treatments were not conducted; (6) studies must be published in English. Studies were excluded if they met the following criteria. (1) review, meta-analysis and case report; (2) basic experimental researches of HCC and studies unrelated to the NK cells and their receptors/ligands; (3) studies provided inadequate data to evaluate the correlation of NK cells and their receptors/ligands with prognosis. For republished studies, only the studies with the largest sample size were selected; alternatively, the most recent literature and relevant data were collected.

### Data Extraction and Quality Assessment

Eligible study data were extracted independently by two investigators (JSX, ZND). Disagreements could be discussed and resolved with a third investigator (GXM). Baseline characteristics were extracted from the included studies. Only one study had outcome indicator for CSS, which we uniformly classified as OS. OS and DFS/RFS/TTR/PFS were used as endpoints for the meta-analysis. The quality of eligible studies was assessed by the Newcastle–Ottawa Scale (NOS) criteria (30).

### Statistical Analysis

Most of the relevant data from the studies could be directly collected. However, for those studies that did not provide hazard ratios (HRs) and 95% confidence intervals (95% CIs), we obtained estimates from known information using the method of Altman and Tierney (31, 32). Random effects models were applied. P < 0.05 was considered statistically significant. The pooled HR and 95% CI were used to assess the relevance between NK cell level and prognosis of HCC patients. Cochran’s Q test and Higgins’ I^2^ statistic were used to assess the heterogeneity. P-value of heterogeneity > 0.10 and I^2^ < 50% were considered as no significant heterogeneity. At the same time, subgroup analyses were performed by surface marker, source of NK cell, and outcome of patients. Sensitivity analysis was performed by removing each study to test the stability and reliability of the results. Funnel plots, Egger regression asymmetry tests, and Begg rank correlation tests were conducted to check for potential publication bias (33). Stata 16.0 software analysis was applied to all data in this meta-analysis.

## Results

### Literature Search

**Figure 1:**
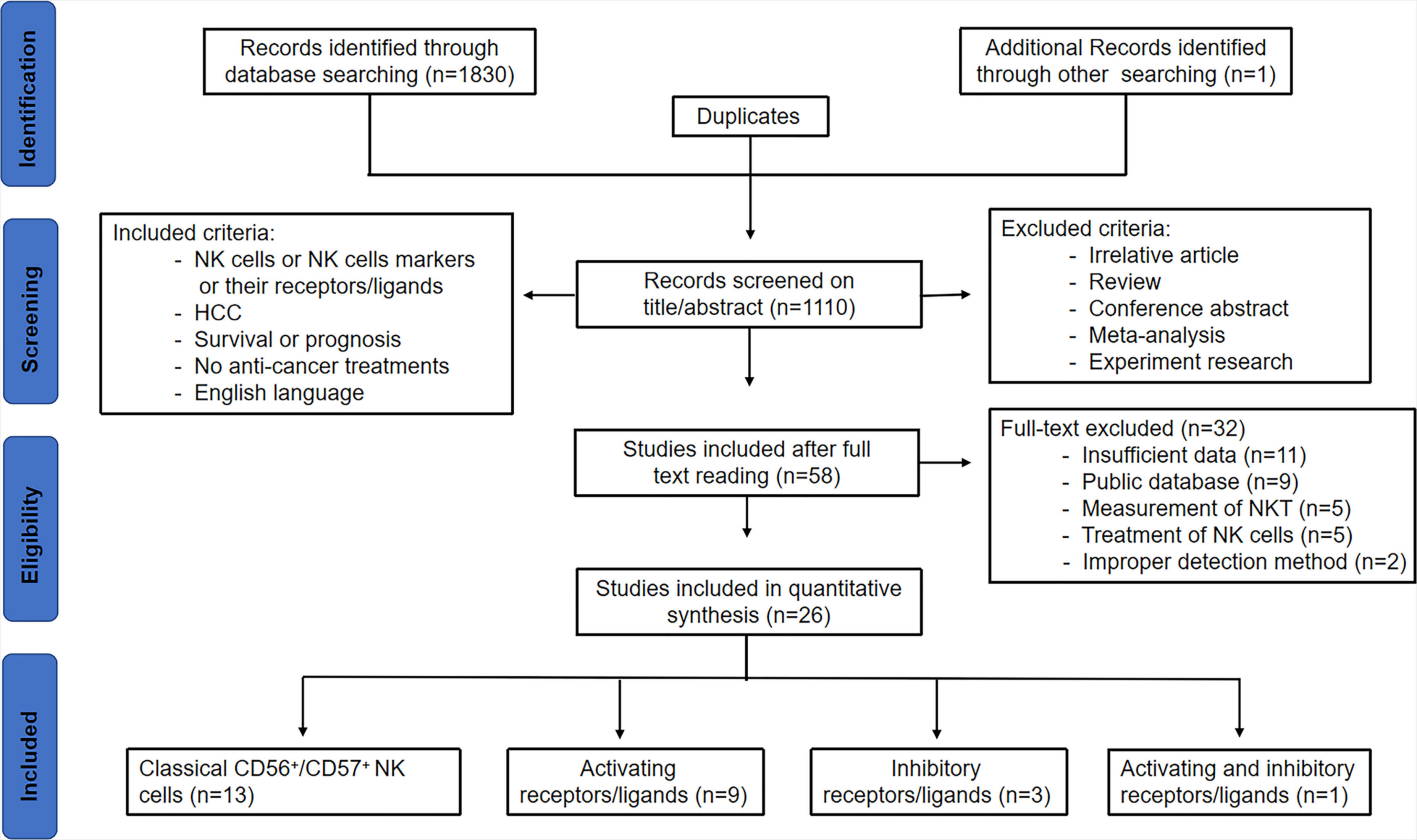
Study flow chart of the data extraction process and selection of studies for meta-analysis.

As shown in **Figure 1** of the flowchart, a total of 1831 records were initially identified. After removing duplicate studies, 1110 studies were retained. After screening for titles and abstracts, 1052 studies were excluded. After reviewing the remaining 58 studies through full text, 32 studies were excluded due to insufficient data (11), public database (9), measurement of NKT (5), improper detection method including Ficoll separation or radiotaged NK-sensitive K-562 cell separation (2), and treatment of NK cells (5), including intravenous infusion of NK cells alone or combined with other modalities, such as radiofrequency ablation and irreversible electroporation. Finally, 26 studies were included in this analysis, including 13 on classical NK cells, 9 on activating receptors/ligands, 3 on inhibitory receptors/ligands, and 1 on both activating and inhibitory receptors/ligands.

### The Basic Characteristics of Included Studies About NK Cells

**Table 1** summarized the basic characteristics of the included studies about NK cells. Sample sizes of the eligible studies ranged from 36 to 258, for a total of 1711, and these studies were conducted primarily in two countries: twelve in China and one in Italy. Seven studies reported on CD56^+^ NK cells, 4 on CD57^+^ NK cells, and 2 on NK cells. Among these studies, 5 studies detected NK cells in peripheral blood, and 8 detected intratumor NK cells. In total, 12 studies mentioned the correlation between NK cell levels and OS (20–22, 25–27, 34–37, 39, 40). 9 studies mentioned the correlation between NK cells levels and DFS/RFS/TTR/PFS (20, 25–27, 35, 36, 38–40). NOS score > 6 was defined as high quality, and ≤ 6 was defined as low quality (**Supplementary Table 1**).

**Table 1:** The characteristics of all included eligible studies about NK cells.

Author	Year	Country	Sample size	Male/Female	Measurement	Marker	Treatment	Source	Tumor stage	VS	Numberof VS	Divide	Outcome	Follow-up times	Score
Zhuang et al. (34)	2019	China	78	64/14	NA	CD56	mixed+SBRT	Peripheral blood	NA	High/Low	NA	cutoff value	OS;PFS	median:32 (4.1-80) month	7
Hu et al. (25)	2021	China	182	NA	Immuno histochemistry	CD57	resection	Intratumor	I-IV	High/Low	NA	NA	OS;TTR	until 30/06/2016	7
Lin et al. (35)	2013	China	132	NA	Immuno histochemistry	CD56	resection	Intratumor	I-III	High/Low	NA	cutoff value	OS;DFS	total:72 month	8
Wu et al. (20)	2013	China	256	115/15	Immuno histochemistry	CD57	resection/RFA	Intratumor	I-IV	High/Low	126/130	median	OS;DFS	NA	9
Tao et al. (26)	2020	China	258	NA	Immuno histochemistry	CD56	resection	Intratumor	I-III	High/Low	129/129	median	OS;TTR	NA	9
Chew et al. (22)	2012	China	36	NA	Immuno histochemistry	CD56	resection	Intratumor	I-IV	High/Low	NA	median	OS	median:3.94 (0.9-5.5) year	6
Zhao et al. (21)	2014	China	163	131/32	Immuno histochemistry	CD57	resection	Intratumor	NA	High/Low	82/81	median	OS	total:>60 month	9
Gao et al. (36)	2012	China	206	NA	Immuno histochemistry	CD57	liver transplantation	Intratumor	I-III	High/Low	NA	median	CSS;RFS	median:48.1 (3.4- 111.9) month	8
Cariani et al. (23)	2016	Italy	70	41/29	Flow cytometry	NK cells	resection/RFA	Peripheral blood	NA	High/Low	NA	median	OS;TTR	median OS:64 month; median TTR:16.5 month	7
Pan et al. (37)	2014	China	121	NA	Flow cytometry	CD56	resection+CIK	Peripheral blood	NA	High/Low	60/61	median	OS	until 31/12/2012	9
Liu et al. (38)	2021	China	100	NA	Immuno histochemistry	CD56	resection	Intratumor	NA	Positive/ Negative	31/68	score	RFS	until:20/06/2020	6
Pan et al. (27)	2020	China	48	39/9	Flow cytometry	CD56	resection+CIK	Peripheral blood	NA	High/Low	24/24	median	OS;RFS	total:>60 month	7
Che et al. (39)	2014	China	61	NA	Flow cytometry	NK cells	resection	Peripheral blood	NA	High/Low	33/28	median	OS;PFS	total:36 month	6

SBRT, Stereotactic body radiation therapy; RFA, Radiofrequency ablation; CIK, Cytokine-induced killer; Mixed, TACE or RFA or PEI or surgery or no treatment; TAE, Transcatheter arterial embolization; NK, Natural killer; OS, Overall survival; DFS, Disease-free survival; RFS, Recurrence-free survival; TTR, Time-to recurrence; PFS, Progression-free survival; CSS, Cancer-specific survival.

NA, Not available.

### Prognostic Value of NK Cells in Patients With HCC

**Figure 2:**
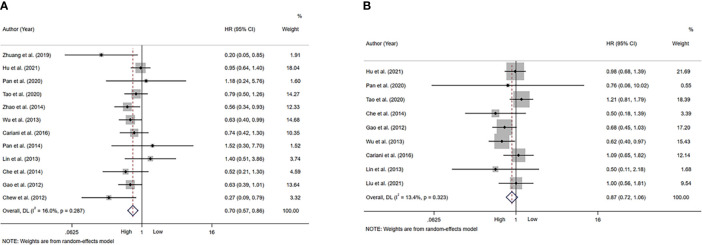
Forest plot of NK cells in HCC. **(A)** Forest plot of NK cells and OS in HCC. **(B)** Forest plot of NK cells and DFS/RFS/TTR/PFS in HCC. CI, Confidence interval; HR, Hazard ratio; HCC, Hepatocellular carcinoma; NK, Natural killer; OS, Overall survival; DFS, Disease-free survival; RFS, Recurrence-free survival; TTR, Time-to recurrence; PFS, Progression-free survival.

**Figure 3:**
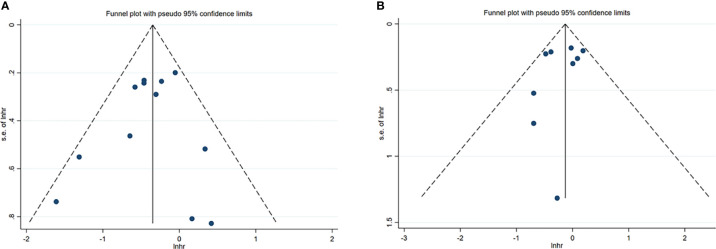
Funnel plots of NK cells in HCC. **(A)** Funnel plots of HR for OS of NK cells. **(B)** Funnel plots of HR for DFS/RFS/TTR/PFS of NK cells. CI, Confidence interval; HR, Hazard ratio; HCC, Hepatocellular carcinoma; NK, Natural killer; OS, Overall survival; DFS, Disease-free survival; RFS, Recurrence-free survival; TTR, Time-to recurrence; PFS, Progression-free survival.

A total of 12 studies, involving 1611 patients, investigated the prognostic value of NK cells for OS. The pooled results from the 12 comparative studies were significant (HR=0.70, 95%CI 0.57-0.86, p=0.001), and the data were not heterogeneous (I^2 =^ 16.0%, P=0.287; **Figure 2A**). No bias was observed in the funnel plot (**Figure 3A**). In order to better understand the prognostic value of NK cells, we further performed subgroup analysis according to the marker and source of NK cells (**Table 2**). In subgroup analysis of CD57^+^ NK cells, the pooled results from 4 comparative studies were significant (HR=0.70, 0.55-0.89, P=0.004), while it was insignificant in CD56^+^ NK cells (HR=0.69, 95%CI 0.38-1.25, P=0.224; **Supplementary Figure 1**). Compared to peripheral NK cells (HR=0.66, 95%CI 0.41-1.06, P=0.088), the level of intratumor NK cells had better prognostic value (HR=0.71, 95%CI 0.55-0.90, P=0.005) for HCC patients (**Supplementary Figure 2**).

**Table 2:** Subgroup meta-analysis of the prognostic role of NK cells in HCC.

Factor	No. of study	No. of patients	HR (95%CI)	P-value	Heterogeneity	
					I^2^(%)	P-value
**OS**						
Total	12	1611	0.70 (0.57-0.86)	0.001	16.0	0.287
**Marker**						
CD56	6	673	0.69 (0.38-1.25)	0.224	45.6	0.102
CD57	4	807	0.70 (0.55-0.89)	0.004	12.8	0.328
NK cells	2	131	0.67 (0.41-1.08)	0.103	0.0	0.532
**Source**						
Peripheral blood	5	378	0.66 (0.41-1.06)	0.088	12.0	0.337
Intratumor	7	1233	0.71 (0.55-0.90)	0.005	29.0	0.207
**DFS/RFS/TTR/PFS**						
Total	9	1313	0.87 (0.72-1.06)	0.164	13.4	0.323
**Marker**						
CD56	4	538	1.09 (0.79-1.50)	0.602	0.0	0.689
CD57	3	644	0.76 (0.57-1.01)	0.059	32.3	0.228
NK cells	2	131	0.84 (0.41-1.73)	0.641	43.8	0.182
**Source**						
Peripheral blood	3	179	0.93 (0.59-1.46)	0.746	0.0	0.406
Intratumor	6	1134	0.86 (0.68-1.09)	0.213	32.1	0.195
**Outcome**						
DFS	2	388	0.61 (0.40-0.93)	0.022	0.0	0.784
RFS	3	354	0.77 (0.55-1.08)	0.134	0.0	0.568
TTR	3	510	1.08 (0.85-1.36)	0.543	0.0	0.737
PFS	1	61	0.50 (0.18-1.39)	0.185	/	/

HCC, Hepatocellular carcinoma; NK, Natural killer; CI, Confidence interval; HR, Hazard ratio; OS, Overall survival; DFS, Disease-free survival; TTR, Time-to recurrence; RFS, Recurrence-free survival; PFS, Progression-free survival.

A total of 9 studies, involving 1313 patients, investigated the prognostic value of NK cells for DFS/RFS/TTR/PFS. The pooled results from the 9 comparative studies were not significant (HR=0.87, 95%CI 0.72-1.06, P=0.164), and the data were not heterogeneous (I^2 =^ 13.4%, P=0.323; **Figure 2B**). No bias was observed in the funnel plot (**Figure 3B**). We also performed subgroup analysis to better understand prognostic value of NK cells (**Table 2**). The pooled HR (95%CI) for CD57^+^ NK cells and CD56^+^ NK cells was 0.76 (0.57-1.01, P=0.059) and 1.09 (0.79-1.50, P=0.602), respectively (**Supplementary Figure 3**). For NK cells derived from peripheral blood and intratumor, the pooled HR (95%CI) was 0.93 (0.59-1.46, P=0.746) and 0.86 (0.68-1.09, P=0.213), respectively (**Supplementary Figure 4**). In addition, in subgroup analysis of outcome, we found that high NK cells levels could be a good predictor for DFS (HR=0.61, 95%CI 0.40-0.93, P=0.022), but not for RFS, TTR and PFS (**Supplementary Figure 5**).

### Prognostic Value of Activating Receptors/Ligands on NK Cells

**Table 3** summarized the outcomes of 3 studies that reported on NKp30^+^ NK cells. One study mentioned that high NKp30^+^ NK cell level was associated with better survival (41), while another study reported no effect of NKp30^+^ NK cell level on patient outcome (43). Other study suggested that high NKp30^+^ NK cell level was associated with good PFS, but not with OS (42). In addition, one study investigated the prognostic role of NKG2D. They concluded that low frequency of circulating NKG2D^+^CD56^dim^ NK cells one month after hepatectomy may predict a poor prognosis for patients with HBV-related HCC (23).

**Table 3:** The characteristics of included studies about the NK cells activating receptors and their ligands.

	Marker	Author	Year	Country	Sample size	Measure ment	Treatment	Source	Tumor stage	VS	Number of VS	Divide	Outcome	P-value	Follow-up times
Activating receptors of the NK cells	NKp30	Chew et al. (41)	2010	Singapore	61	Immuno histochemistry	resection	Intratumor	I-III	High/Low	NA	median	OS	HR (95%CI) 0.34 (0.13,0.85) P=0.0144	median: 2.56 (0.02-9.11) year
	NKp30	Li et al. (42)	2021	China	25	Flow cytometry	untreated	Peripheral blood	NA	High/Low	16/9	cutoff value	OS;PFS	Log-rank test P=0.279; Log-rank test P=0.016	NA
	NKG2D	Gao et al. (23)	2016	China	20	Flow cytometry	resection	Peripheral blood	I-III	High/Low	10/10	median	OS;RFS	Log-rank test P=0.014; Log-rank test P=0.010	until:2014.11
	NKp30	Rochigneux et al. (43)	2019	France	57	Flow cytometry	RFA	Peripheral blood	NA	High/Low	28/29	median	PFS	HR (95%CI) 0.61 (0.29,1.29) P=0.20	until:12/2016
Ligands of the NK cells activating receptors	soluble MICA	Li et al. (44)	2013	China	60	ELISA	TACE	serum	III/IV	High/Low	28/32	median	OS	HR (95%CI) 1.47 (1.01,1.95) P<0.001	until:31/08/2010
	B7-H6	Qiu et al. (45)	2021	China	90	Immuno histochemistry	resection	Intratumor	I/II	High/Low	33/57	mean-H score	OS;DFS	HR (95%CI) 0.47 (0.24,0.93) P=0.029; HR (95%CI) 0.72 (0.36,1.43) P=0.1013	total:>60 month
	MICA	Zhang et al. (28)	2014	China	143	Immuno histochemistry	resection	Intratumor	I-IV	High/Low	NA	NA	OS;RFS	HR (95%CI) 0.91 (0.49,1.69) P=0.774; HR (95%CI) 1.43 (0.90,2.27) P=0.135	until:08/2013
	MICA/B	Fang et al. (46)	2014	China	96	Immuno histochemistry	resection	Intratumor	I-IV	High/Low	75/21	MICA/B expression score	OS	HR (95%CI) 0.32 (0.11,0.92) P<0.001	until:08/2012
	ULBP1	Kamimura et al. (47)	2012	Japan	54	Immuno histochemistry	untreated/ resection	Intratumor	NA	Positive/Negative	25/47	expression	OS;RFS	HR (95%CI) 0.72 (0.09,5.70) P=0.120; HR (95%CI) 0.2 (0.06,0.65) P=0.006	NA
	ULBP1	Easom et al. (48)	2020	England	72	ELISA	untreated	serum	NA	High/Low	NA	NA	OS	HR (95%CI) 2.11 (1.02,4.02) P=0.0029	NA

NK, Natural killer; CI, Confidence interval; HR, Hazard ratio; RFA, Radiofrequency ablation; ELISA, Enzyme-linked immunosorbent assay; TACE, Transcatheter arterial chemoembolization; OS, Overall survival; DFS, Disease-free survival; RFS, Recurrence-free survival; PFS, Progression-free survival; NA, Not available.

**Figure 4:**
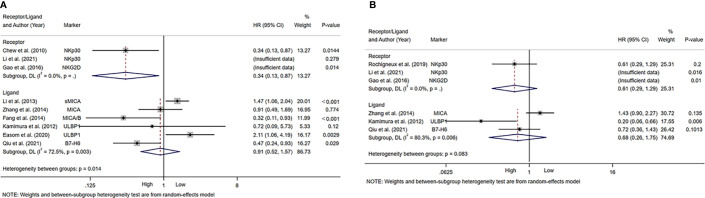
Forest plot of NK cells activating receptors/ligands in HCC. **(A)** Forest plot of NK cells activating receptors/ligands and OS in HCC. **(B)** Forest plot of NK cells activating receptors/ligands and DFS/RFS/PFS in HCC. CI, Confidence interval; HR, Hazard ratio; HCC, Hepatocellular carcinoma; NK, Natural killer; OS, Overall survival; DFS, Disease-free survival; RFS, Recurrence-free survival; PFS, Progression-free survival.

A total of 6 studies reported on ligands of NK cell activating receptors, including MICA, MICB, soluble MICA (sMICA), ULBP1 and B7-H6 (28, 44–48). The pooled HR (95%CI) for OS and DFS/RFS/PFS was 0.91 (0.52-1.57, P=0.726; **Figure 4A**) and 0.68 (0.26-1.75, P=0.422; **Figure 4B**), respectively.

### Prognostic Value of Inhibitory Receptors/Ligands on NK Cells

**Figure 5:**
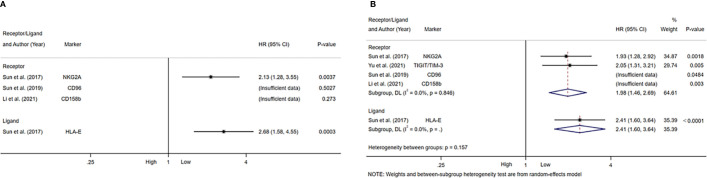
Forest plot of NK cells inhibitory receptors/ligands in HCC. **(A)** Forest plot of NK cells inhibitory receptors/ligands and OS in HCC. **(B)** Forest plot of NK cells inhibitory receptors/ligands and DFS/PFS in HCC. CI, Confidence interval; HR, Hazard ratio; HCC, Hepatocellular carcinoma; NK, Natural killer; OS, Overall survival; DFS, Disease-free survival; PFS, Progression-free survival.

**Table 4** summarized the outcomes of 4 studies that mentioned NK cell inhibitory receptors, including NKG2A, CD96, CD158b, TIGIT and TIM-3 (24, 42, 49, 50). They all concluded that high level of NK cell inhibitory receptors predicted increased recurrence of HCC patients. Sun and Li et al. suggested that the level of NK cell inhibitory receptors was not associated with survival of HCC patients, while other studies revealed that high NK cell inhibitory receptors level predicted poor survival of HCC patients. One study found that intratumor level of HLA-E was increased, and high HLA-E level was correlated with poor prognosis of HCC patients (**Figures 5A, B**).

**Table 4:** The characteristics of included studies about the NK cells inhibitory receptors and their ligands.

	Marker	Author	Year	Country	Sample size	Measurement	Treatment	Source	Tumor stage	VS	Number of VS	Divide	Outcome	P-value	Follow-up times
Inhibitory receptors of the NK cells	NKG2A	Sun et al. (24)	2017	China	177	Immune histochemistry	resection	Intratumor	NA	High/Low	68/109	cutoff value	OS;DFS	HR (95%CI) 2.13 (1.28,3.56) P=0.0037; HR (95%CI) 1.93 (1.28,2.93) P=0.0018	median OS:1299.7 ± 1974.2 day; median DFS:980.0 ± 1796.1 day
	CD96	Sun et al. (49)	2019	China	236	Flow cytometry	resection	Intratumor	NA	High/Low	NA	cutoff value	OS;DFS	Log-rank test P=0.5027; Log-rank test P=0.0484	NA
	CD158b	Li et al. (43)	2021	China	13	Flow cytometry	SBRT	Peripheral blood	NA	High/Low	5/8	cutoff value	OS;PFS	Log-rank test P=0.273; Log-rank test P=0.003	NA
	TIGIT TIM-3	Yu et al. (50)	2021	China	133	Flow cytometry	palliative/minimally invasive/resection	Peripheral blood	NA	High/Low	65/68	cutoff value	PFS	HR (95%CI) 2.05 (1.24,3.04) P=0.005	NA
Ligands of the NK cells inhibitory receptors	HLA-E	Sun et al. (49)	2017	China	177	Immune histochemistry	resection	Intratumor	NA	High/Low	79/98	cutoff value	OS;DFS	HR (95%CI) 2.68 (1.58,4.56) P=0.0003; HR (95%CI) 2.41 (1.60,3.64) P<0.0001	median OS:1299.7 ± 1974.2 day; median DFS:980.0 ± 1796.1 day

NK, Natural killer; CI, Confidence interval; HR, Hazard ratio; SBRT: Stereotactic body radiation therapy; OS, Overall survival; DFS, Disease-free survival; PFS, Progression-free survival; NA, Not available.

### Assessment of Sensitivity Analysis and Publication Bias

**Figure 6:**
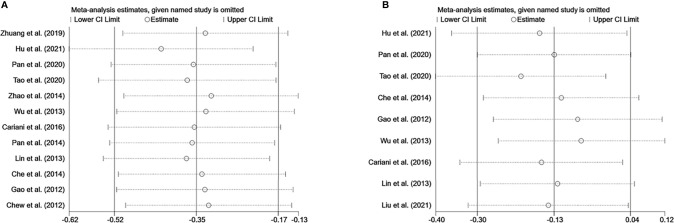
Sensitivity analysis of NK cells. **(A)** Sensitivity analysis for OS of NK cells. **(B)** Sensitivity analysis for DFS/RFS/TTR/PFS of NK cells. CI, Confidence interval; HR, Hazard ratio; NK, Natural killer; OS, Overall survival; DFS, Disease-free survival; RFS, Recurrence-free survival; TTR, Time-to recurrence; PFS, Progression-free survival.

**Figure 7:**
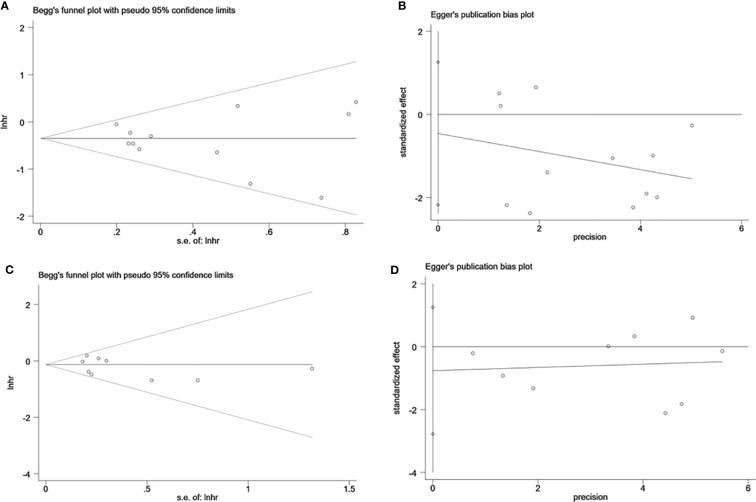
Evaluation of publication bias of NK cells using Begg’s test and Egger’s test. **(A)** Begg’s test for OS of NK cells, P=0.732. **(B)** Egger’s test for OS of NK cells, P=0.564. **(C)** Begg’s test for DFS/RFS/TTR/PFS of NK cells, P=0.602. **(D)** Egger’s test for DFS/RFS/TTR/PFS of NK cells, P=0.401. lnhr, the ln of HR; s.e., standard error; NK, Natural killer; OS, Overall survival, DFS, Disease-free survival; RFS, Recurrence-free survival; TTR, Time-to recurrence; PFS, Progression-free survival.

Sensitivity analysis was performed to evaluate the stability of NK cells for predicting survival and recurrence of HCC patients. After removing any of the studies, the results did not exceed the 95% CI range of the pooled results (**Figures 6A, B**). Begg’s test and Egger’s linear regression test were used to assess whether there was potential publication bias in this meta-analysis. The results showed that no apparent publication bias for the analysis was found between NK cells and OS (Begg’s test: P=0.732, **Figure 7A**; Egger’s test: P=0.564, **Figure 7B**). Similarly, no significant publication bias was found for DFS/RFS/TTR/PFS analysis (Begg’s test: P=0.602, **Figure 7C**; Egger’s test: P=0.401, **Figure 7D**).

## Discussion

NK cells are innate lymphocytes that can kill virus-infected or cancer cells, and have a vital role in early hepatocarcinogenesis (51, 52). Different to T cells which require somatic gene rearrangement to produce highly antigen-specific receptors (53), NK cells are innately equipped with germline-encoded activating and inhibitory receptors that can directly determine whether NK cells are activated or inhibited (12, 54). NK cells can deliver cytotoxic granules, secrete effector cytokines, and are involved in death receptor induced apoptosis (55). NK cells can also rapidly produce cytokines with anti-tumor effects, such as IFN-γ, to exert their killing effects in the early stage of disease (56). In addition, NK cells can bind to target cells through surface CD16 and kill them through exerting antibody-dependent cell-mediated cytotoxicity (ADCC) (57). These results imply that NK cells play an essential role in the body’s immune process in the defense against HCC.

In this study, we found that high NK cell level could predict better survival for patients with HCC. Similar results were previously reported in a meta-analysis of solid tumors (58). In subgroup analysis, CD57^+^ NK cells had better prognostic value over CD56^+^ NK cells. On the one hand, it may be that CD56^+^ NK cells, accounting for the majority of circulating NK cells, also expressed inhibitory molecules, which may strive for a dynamic balance between activating and inhibitory molecules. Moreover, lower IFN-γ production was also described in HCC, in accordance with the decreased cytotoxicity of NK cells (59). On the other hand, acquisition of CD57 represents a shift toward a higher cytotoxic capacity, greater responsiveness to signaling *via* CD16 and natural NCRs (60). The same result was also observed in Hu et al.’s study (61). In addition, compared to peripheral NK cells, NK cells from intratumor had better prognostic value for prognosis of HCC patients, possibly because NK cells are abundant in human liver.

NK cells express activating and inhibitory receptors in order to perceive signals and display their activity. Depending on the received signal, NK cells can be activated or restricted (62). In this study, activating receptors/ligands mainly contain NKG2D, NKp30, and their ligands. NKG2D ligands mainly consist of ULBPs and MICA/B. MICA/B molecules expressed on HCC cells are recognized by NKG2D to induce ubiquitination-mediated endocytosis of the NKG2D-DAP10 complex, thereby activating NK cells to kill HCC (46, 57). NKp30 contains two ligands. One is BAT3, a nuclear protein that induces apoptosis in target cells by interacting with P53. The other is B7-H6, a newly discovered member of the B7 family that is expressed on the surface of tumor cells (45). B7-H6/NKp30 pathway is involved in the NK cell-mediated immune responses, and NK cells can recognize and eliminate B7-H6-expressing tumors, including HCC. However, tumors can also impair NK cell function by shedding B7-H6 membranes or decreasing NKp30 expression, leading to tumor immune escape and tumor progression (63). In addition, hypoxia, some soluble forms of NCRs ligands, or soluble factors produced by tumor/tumor-associated cells, can induce a decrease in both NCR expression and function (10, 64, 65), and protect tumor from NK cell-mediated cytotoxicity (44, 66). Until now, the prognostic value of activating receptors remained inconsistent from different studies and deserved further investigation.

Inhibitory receptors/ligands mainly contain NKG2A, CD96, CD158b, TIGIT and TIM-3, and HLA-E. The NKG2A is expressed approximately in half of the peripheral blood NK cells, and is also expressed on CD8^+^ T cells (67). The inhibitory signals induced by NKG2A engagement can result in decreased capacity of NK cells and CD8^+^ T cells to lyse target cells (68), and enhanced expression of the HLA-E on tumor may result in resistance and immune escape by binding to NKG2A (68). Therefore, blocking the interaction of NKG2A with HLA-E has shown promising therapeutic effects in animal study (69). In addition, there is increasing evidence that PD-1 is also expressed on the surface of NK cells and exerts a suppressive function on T cell responses (16). In this study, though we found that inhibitory receptors of NK cells may be a good predictor for recurrence of HCC, however, their prognostic value in predicting survival was unclear. Therefore, more high-quality prospective studies are needed to explore the prognostic value of NK cells and their receptors/ligands for HCC.

The strength of this study is that it explored for the first time the prognostic value of NK cells and their receptors/ligands in HCC. Almost all relevant articles that could be collected were included and a comprehensive analysis was provided. However, the following limitations should also be considered. First, some of the data were not obtained directly from the included studies. HRs and 95% CIs were calculated using survival curves or 95% CIs were calculated from known P values and HRs, which may result in data inaccuracy to some extent. Second, although most studies used median as the cut-off value for NK cells level, these values were complex and related to the clinicopathological characteristics of the HCC patients. Third, although DFS/RFS/TTR/PFS of HCC patients are considered as composite outcome indicators, there are still slightly difference between them. Fourth, NK cells receptors/ligands are diverse. The pooled results may exist in bias to some extent. Fifth, this was an aggregate data rather than an individual data meta-analysis, and the data were various between studies, which somehow diminished the significance of the study. Finally, some surface markers were expressed not only in NK cells, but also in other immune cells, such as T cells, Dendritic cells (DCs), etc. Moreover, liver NK cells are composed of several subsets including conventional, type 1 innate lymphoid cells (ILC1) like, and liver-resident NK cells. All of them can express similar molecules and potentially be responsible for the clinical benefit observed (70).

## Conclusions

In summary, we concluded that NK cells could be a good predictor for survival of HCC. More importantly, CD57^+^ NK cells may have better prognostic value over CD56^+^ NK cells, and intratumor NK cells have better prognostic value over peripheral NK cells. Inhibitory receptors of NK cell may be a good predictor for recurrence of HCC, but the value of activating and inhibitory receptors in predicting the survival of HCC was unclear. More high-quality prospective studies are essential to evaluate the prognostic value of NK cells and their receptors/ligands for HCC.

## Data Availability Statement

The original contributions presented in the study are included in the article/**Supplementary Material**. Further inquiries can be directed to the corresponding author.

## Author Contributions

J-SX and TL were responsible for designing the study. J-SX, Z-ND, and G-XM conducted the systematic search and performed the screening. J-SX, Z-ND, G-XM, L-JY, HL, H-CL, S-YY, B-WT, J-GH, Z-RD, Z-QC, and D-XW primarily performed the quality assessment as well as supervision. J-SX analyzed, interpreted the data, and drafted the manuscript. TL revised the manuscript. All data and material analyzed during this study were included in this article. All authors have read and approved the final version of the manuscript.

## Funding

This work was supported by the grants from the Taishan Scholars Program for Young Expert of Shandong Province (Grant No. tsqn20161064), National Natural Science Foundation of China (Grant No. 82073200 & 81874178), funds for Independent Cultivation of Innovative Team from Universities in Jinan (Grant No. 2020GXRC023), and Major basic research of Shandong Provincial Natural Science Foundation (Grant No. ZR202105070027).

## Conflict of Interest

The authors declare that the research was conducted in the absence of any commercial or financial relationships that could be construed as a potential conflict of interest.

## Publisher’s Note

All claims expressed in this article are solely those of the authors and do not necessarily represent those of their affiliated organizations, or those of the publisher, the editors and the reviewers. Any product that may be evaluated in this article, or claim that may be made by its manufacturer, is not guaranteed or endorsed by the publisher.

## Glossary

**Table:**

NK	Natural killer
HCC	Hepatocellular carcinoma
HRs	Hazard ratios
CI	Confidence interval
NKG2D	NK group 2 member D
NCRs	Natural cytotoxic receptors
IL	Interleukin
IFNR	Interferon receptor
TGF-βR	Tumor growth factor-β receptor
BAT3	HLA-B-associated transcript 3
BAG6	Bcl-2-associated athanogene 6
MLL5	Mixed lineage leukemia 5
PCNA	Proliferating cell nuclear antigen
B7-H6	B7 homolog 6
MIC	Major histocompatibility complex class I chain-related protein
ULBPs	UL16-binding proteins
KIRs	Killer immunoglobulin-like receptors
TIM-3	T cell immunoglobulin domain and mucin domain-3
TIGIT	T cell immunoglobulin and immunoreceptor tyrosine-based inhibitory motif domain
PD-1	Programmed cell death-1
PD-L1	Programmed cell death ligand-1
LAG3	Lymphocyte activation gene-3
LAIRs	Leukocyte-associated immunoglobulin-like receptors
A2AR	Adenosine 2A receptor
ILTs	Immunoglobulin-like transcripts
HLA-E	Histocompatibility leucocyte antigen E
MHC-I	Major histocompatibility complex class I
PRISMA	Preferred Reporting Items for Systematic reviews and Meta-Analyses
OS	Overall survival
CSS	Cancer-specific survival
DFS	Disease-free survival
RFS	Recurrence-free survival
TTR	Time-to recurrence
PFS	Progression-free survival
NOS	Newcastle–Ottawa Scale
sMICA	Soluble MICA
ADCC	Antibody-dependent cell-mediated cytotoxicity
DCs	Dendritic cells
ILC1	Type 1 innate lymphoid cells

## References

[1] Sung H Ferlay J Siegel RL Laversanne M Soerjomataram I Jemal A . Global Cancer Statistics 2020: Globocan Estimates of Incidence and Mortality Worldwide for 36 Cancers in 185 Countries. CA: Cancer J Clin (2021) 71(3):209–49. doi: 10.3322/caac.21660 33538338

[2] Yang JD Hainaut P Gores GJ Amadou A Plymoth A Roberts LR . A Global View of Hepatocellular Carcinoma: Trends, Risk, Prevention and Management. Nat Rev Gastroenterol Hepatol (2019) 16(10):589–604. doi: 10.1038/s41575-019-0186-y 31439937 PMC6813818

[3] Vivier E Tomasello E Baratin M Walzer T Ugolini S . Functions of Natural Killer Cells. Nat Immunol (2008) 9(5):503–10. doi: 10.1038/ni1582 18425107

[4] Mikulak J Bruni E Oriolo F Di Vito C Mavilio D . Hepatic Natural Killer Cells: Organ-Specific Sentinels of Liver Immune Homeostasis and Physiopathology. Front Immunol (2019) 10:946. doi: 10.3389/fimmu.2019.00946 31114585 PMC6502999

[5] Caligiuri MA . Human Natural Killer Cells. Blood (2008) 112(3):461–9. doi: 10.1182/blood-2007-09-077438 PMC2481557 18650461

[6] Campbell KS Hasegawa J . Natural Killer Cell Biology: An Update and Future Directions. J Allergy Clin Immunol (2013) 132(3):536–44. doi: 10.1016/j.jaci.2013.07.006 PMC3775709 23906377

[7] Solana R Tarazona R Gayoso I Lesur O Dupuis G Fulop T . Innate Immunosenescence: Effect of Aging on Cells and Receptors of the Innate Immune System in Humans. Semin Immunol (2012) 24(5):331–41. doi: 10.1016/j.smim.2012.04.008 22560929

[8] Huntington ND Cursons J Rautela J . The Cancer-Natural Killer Cell Immunity Cycle. Nat Rev Cancer (2020) 20(8):437–54. doi: 10.1038/s41568-020-0272-z 32581320

[9] Hammer Q Rückert T Romagnani C . Natural Killer Cell Specificity for Viral Infections. Nat Immunol (2018) 19(8):800–8. doi: 10.1038/s41590-018-0163-6 30026479

[10] Sivori S Vacca P Del Zotto G Munari E Mingari MC Moretta L . Human Nk Cells: Surface Receptors, Inhibitory Checkpoints, and Translational Applications. Cell Mol Immunol (2019) 16(5):430–41. doi: 10.1038/s41423-019-0206-4 PMC6474200 30778167

[11] Liu H Wang S Xin J Wang J Yao C Zhang Z . Role of Nkg2d and Its Ligands in Cancer Immunotherapy. Am J Cancer Res (2019) 9(10):2064–78. PMC6834480 31720075

[12] Martinet L Smyth MJ . Balancing Natural Killer Cell Activation Through Paired Receptors. Nat Rev Immunol (2015) 15(4):243–54. doi: 10.1038/nri3799 25743219

[13] Li J Tao L Wang X . Cytotoxic Immune Cell-Based Immunotherapy for Hepatocellular Carcinoma. Hepatoma Res (2020) 6:15. doi: 10.20517/2394-5079.2019.34

[14] Kalathil SG Thanavala Y . Natural Killer Cells and T Cells in Hepatocellular Carcinoma and Viral Hepatitis: Current Status and Perspectives for Future Immunotherapeutic Approaches. Cells (2021) 10(6):1332. doi: 10.3390/cells10061332 34071188 PMC8227136

[15] Pegram HJ Andrews DM Smyth MJ Darcy PK Kershaw MH . Activating and Inhibitory Receptors of Natural Killer Cells. Immunol Cell Biol (2011) 89(2):216–24. doi: 10.1038/icb.2010.78 20567250

[16] Quatrini L Mariotti FR Munari E Tumino N Vacca P Moretta L . The Immune Checkpoint Pd-1 in Natural Killer Cells: Expression, Function and Targeting in Tumour Immunotherapy. Cancers (2020) 12(11):3285. doi: 10.3390/cancers12113285 PMC7694632 33172030

[17] Eugène J Jouand N Ducoin K Dansette D Oger R Deleine C . The Inhibitory Receptor Cd94/Nkg2a on Cd8(+) Tumor-Infiltrating Lymphocytes in Colorectal Cancer: A Promising New Druggable Immune Checkpoint in the Context of Hlae/B2m Overexpression. Modern Pathol (2020) 33(3):468–82. doi: 10.1038/s41379-019-0322-9 31409873

[18] Hiraoka N Ino Y Hori S Yamazaki-Itoh R Naito C Shimasaki M . Expression of Classical Human Leukocyte Antigen Class I Antigens, Hla-E and Hla-G, Is Adversely Prognostic in Pancreatic Cancer Patients. Cancer Sci (2020) 111(8):3057–70. doi: 10.1111/cas.14514 PMC7419048 32495519

[19] Zeestraten EC Reimers MS Saadatmand S Goossens-Beumer IJ Dekker JW Liefers GJ . Hla-E and Hla-G Predicts Prognosis in Colon Cancer Patients. Br J Cancer (2014) 110(2):459–68. doi: 10.1038/bjc.2013.696 PMC3899753 24196788

[20] Wu Y Kuang DM Pan WD Wan YL Lao XM Wang D . Monocyte/Macrophage-Elicited Natural Killer Cell Dysfunction in Hepatocellular Carcinoma Is Mediated by Cd48/2b4 Interactions. Hepatol (Baltimore Md) (2013) 57(3):1107–16. doi: 10.1002/hep.26192 23225218

[21] Zhao J-J Pan Q-Z Pan K Weng D-S Wang Q-J Li J-J . Interleukin-37 Mediates the Antitumor Activity in Hepatocellular Carcinoma: Role for Cd57+Nk Cells. Sci Rep (2014) 4:5177. doi: 10.1038/srep05177 24898887 PMC4046124

[22] Chew V Chen J Lee D Loh E Lee J Lim KH . Chemokine-Driven Lymphocyte Infiltration: An Early Intratumoural Event Determining Long-Term Survival in Resectable Hepatocellular Carcinoma. Gut (2012) 61(3):427–38. doi: 10.1136/gutjnl-2011-300509 PMC3273680 21930732

[23] Gao J Duan Z Zhang L Huang X Long L Tu J . Failure Recovery of Circulating Nkg2d(+)Cd56(Dim)Nk Cells in Hbv-Associated Hepatocellular Carcinoma After Hepatectomy Predicts Early Recurrence. Oncoimmunology (2016) 5(1):e1048061. doi: 10.1080/2162402x.2015.1048061 26942056 PMC4760296

[24] Sun C Xu J Huang Q Huang M Wen H Zhang C . High Nkg2a Expression Contributes to Nk Cell Exhaustion and Predicts a Poor Prognosis of Patients With Liver Cancer. Oncoimmunology (2017) 6(1):e1264562. doi: 10.1080/2162402x.2016.1264562 28197391 PMC5283631

[25] Hu ZQ Xin HY Luo CB Li J Zhou ZJ Zou JX . Associations Among the Mutational Landscape, Immune Microenvironment, and Prognosis in Chinese Patients With Hepatocellular Carcinoma. Cancer Immunol Immunother: CII (2021) 70(2):377–89. doi: 10.1007/s00262-020-02685-7 PMC10992292 32761426

[26] Tao P Hong L Tang W Lu Q Zhao Y Zhang S . Comprehensive Characterization of Immunological Profiles and Clinical Significance in Hepatocellular Carcinoma. Front Oncol (2020) 10:574778. doi: 10.3389/fonc.2020.574778 33552954 PMC7862794

[27] Pan QZ Liu Q Zhou YQ Zhao JJ Wang QJ Li YQ . Cik Cell Cytotoxicity Is a Predictive Biomarker for Cik Cell Immunotherapy in Postoperative Patients With Hepatocellular Carcinoma. Cancer Immunol Immunother: CII (2020) 69(5):825–34. doi: 10.1007/s00262-020-02486-y PMC11027814 32060687

[28] Zhang J Xu Z Zhou X Zhang H Yang N Wu Y . Loss of Expression of Mhc Class I-Related Chain a (Mica) Is a Frequent Event and Predicts Poor Survival in Patients With Hepatocellular Carcinoma. Int J Clin Exp Pathol (2014) 7(6):3123–31. PMC4097291 25031731

[29] Shamseer L Moher D Clarke M Ghersi D Liberati A Petticrew M . Preferred Reporting Items for Systematic Review and Meta-Analysis Protocols (Prisma-P) 2015: Elaboration and Explanation. BMJ (Cli Res ed) (2015) 350:g7647. doi: 10.1136/bmj.g7647 25555855

[30] Stang A . Critical Evaluation of the Newcastle-Ottawa Scale for the Assessment of the Quality of Nonrandomized Studies in Meta-Analyses. Eur J Epidemiol (2010) 25(9):603–5. doi: 10.1007/s10654-010-9491-z 20652370

[31] Tierney JF Stewart LA Ghersi D Burdett S Sydes MR . Practical Methods for Incorporating Summary Time-To-Event Data Into Meta-Analysis. Trials (2007) 8:16. doi: 10.1186/1745-6215-8-16 17555582 PMC1920534

[32] Altman DG Bland JM . How to Obtain the Confidence Interval From a P Value. BMJ (Cli Res ed) (2011) 343:d2090. doi: 10.1136/bmj.d2090 21824904

[33] Egger M Davey Smith G Schneider M Minder C . Bias in Meta-Analysis Detected by a Simple, Graphical Test. BMJ (Cli Res ed) (1997) 315(7109):629–34. doi: 10.1136/bmj.315.7109.629 PMC2127453 9310563

[34] Zhuang Y Yuan BY Chen GW Zhao XM Hu Y Zhu WC . Association Between Circulating Lymphocyte Populations and Outcome After Stereotactic Body Radiation Therapy in Patients With Hepatocellular Carcinoma. Front Oncol (2019) 9:896. doi: 10.3389/fonc.2019.00896 31552194 PMC6748162

[35] Lin SZ Chen KJ Xu ZY Chen H Zhou L Xie HY . Prediction of Recurrence and Survival in Hepatocellular Carcinoma Based on Two Cox Models Mainly Determined by Foxp3+ Regulatory T Cells. Cancer Prev Res (Philadelphia Pa) (2013) 6(6):594–602. doi: 10.1158/1940-6207.Capr-12-0379 23599540

[36] Gao Q Zhou J Wang XY Qiu SJ Song K Huang XW . Infiltrating Memory/Senescent T Cell Ratio Predicts Extrahepatic Metastasis of Hepatocellular Carcinoma. Ann Surg Oncol (2012) 19(2):455–66. doi: 10.1245/s10434-011-1864-3 21792513

[37] Pan K Wang QJ Liu Q Zheng HX Li YQ Weng DS . The Phenotype of Ex Vivo Generated Cytokine-Induced Killer Cells Is Associated With Overall Survival in Patients With Cancer. Tumour Biol (2014) 35(1):701–7. doi: 10.1007/s13277-013-1096-1 23955802

[38] Liu J Kuang S Zheng Y Liu M Wang L . Prognostic and Predictive Significance of the Tumor Microenvironment in Hepatocellular Carcinoma. Cancer Biomarkers: Section A Dis Markers (2021) 32(1):99–110. doi: 10.3233/cbm-203003 PMC12500032 34092607

[39] Che Y-Q Feng L Rong W-Q Shen D Wang Q Yang L . Correlation Analysis of Peripheral Blood T Cell Subgroups, Immunoglobulin and Prognosis of Early Hepatocellular Carcinoma After Hepatectomy. Int J Clin Exp Med (2014) 7(11):4282–90. PMC4276201 25550943

[40] Cariani E Pilli M Barili V Porro E Biasini E Olivani A . Natural Killer Cells Phenotypic Characterization as an Outcome Predictor of Hcv-Linked Hcc After Curative Treatments. Oncoimmunology (2016) 5(8):e1154249. doi: 10.1080/2162402x.2016.1154249 27622055 PMC5007972

[41] Chew V Tow C Teo M Wong HL Chan J Gehring A . Inflammatory Tumour Microenvironment Is Associated With Superior Survival in Hepatocellular Carcinoma Patients. J Hepatol (2010) 52(3):370–9. doi: 10.1016/j.jhep.2009.07.013 19720422

[42] Li TT Sun J Wang Q Li WG He WP Yang RC . The Effects of Stereotactic Body Radiotherapy on Peripheral Natural Killer and Cd3(+)Cd56(+) Nkt-Like Cells in Patients With Hepatocellular Carcinoma. Hepatobil Pancreatic Dis Int: HBPD Int (2021) 20(3):240–50. doi: 10.1016/j.hbpd.2020.12.015 33454220

[43] Rochigneux P Nault JC Mallet F Chretien AS Barget N Garcia AJ . Dynamic of Systemic Immunity and Its Impact on Tumor Recurrence After Radiofrequency Ablation of Hepatocellular Carcinoma. Oncoimmunology (2019) 8(8):1615818. doi: 10.1080/2162402X.2019.1615818 31413924 PMC6682367

[44] Li JJ Pan K Gu MF Chen MS Zhao JJ Wang H . Prognostic Value of Soluble Mica Levels in the Serum of Patients With Advanced Hepatocellular Carcinoma. Chin J Cancer (2013) 32(3):141–8. doi: 10.5732/cjc.012.10025 PMC3845598 22704489

[45] Qiu H Gao S Sun Z Wang J . Dual Role of B7-H6 as a Novel Prognostic Marker in Hepatocellular Carcinoma. APMIS: Acta Pathol Microbiol Immunol Scand (2021) 129(3):105–17. doi: 10.1111/apm.13099 33220098

[46] Fang L Gong J Wang Y Liu R Li Z Wang Z . Mica/B Expression Is Inhibited by Unfolded Protein Response and Associated With Poor Prognosis in Human Hepatocellular Carcinoma. J Exp Clin Cancer Res: CR (2014) 33(1):76. doi: 10.1186/s13046-014-0076-7 25228093 PMC4174668

[47] Kamimura H Yamagiwa S Tsuchiya A Takamura M Matsuda Y Ohkoshi S . Reduced Nkg2d Ligand Expression in Hepatocellular Carcinoma Correlates With Early Recurrence. J Hepatol (2012) 56(2):381–8. doi: 10.1016/j.jhep.2011.06.017 21756848

[48] Easom NJW Marks M Jobe D Gillmore R Meyer T Maini MK . Ulbp1 Is Elevated in Human Hepatocellular Carcinoma and Predicts Outcome. Front Oncol (2020) 10:971. doi: 10.3389/fonc.2020.00971 32656081 PMC7324784

[49] Sun H Huang Q Huang M Wen H Lin R Zheng M . Human Cd96 Correlates to Natural Killer Cell Exhaustion and Predicts the Prognosis of Human Hepatocellular Carcinoma. Hepatol (Baltimore Md) (2019) 70(1):168–83. doi: 10.1002/hep.30347 30411378

[50] Yu L Liu X Wang X Yan F Wang P Jiang Y . Tigit(+) Tim-3(+) Nk Cells Are Correlated With Nk Cell Exhaustion and Disease Progression in Patients With Hepatitis B Virus−Related Hepatocellular Carcinoma. Oncoimmunology (2021) 10(1):1942673. doi: 10.1080/2162402x.2021.1942673 34249476 PMC8244763

[51] Liu S Galat V Galat Y Lee YKA Wainwright D Wu J . Nk Cell-Based Cancer Immunotherapy: From Basic Biology to Clinical Development. J Hematol Oncol (2021) 14(1):7. doi: 10.1186/s13045-020-01014-w 33407739 PMC7788999

[52] Lim O Jung MY Hwang YK Shin EC . Present and Future of Allogeneic Natural Killer Cell Therapy. Front Immunol (2015) 6:286. doi: 10.3389/fimmu.2015.00286 26089823 PMC4453480

[53] Reindl LM Albinger N Bexte T Müller S Hartmann J Ullrich E . Immunotherapy With Nk Cells: Recent Developments in Gene Modification Open Up New Avenues. Oncoimmunology (2020) 9(1):1777651. doi: 10.1080/2162402x.2020.1777651 33457093 PMC7781759

[54] Podhorzer A Machicote A Belén S Lauferman L Imventarza O Montal S . Intrahepatic and Peripheral Blood Phenotypes of Natural Killer and T Cells: Differential Surface Expression of Killer Cell Immunoglobulin-Like Receptors. Immunology (2018) 154(2):261–73. doi: 10.1111/imm.12880 PMC5980203 29247515

[55] Hodgins JJ Khan ST Park MM Auer RC Ardolino M . Killers 2.0: Nk Cell Therapies at the Forefront of Cancer Control. J Clin Invest (2019) 129(9):3499–510. doi: 10.1172/jci129338 PMC6715409 31478911

[56] Zhang S Saha B Kodys K Szabo G . Ifn-Γ Production by Human Natural Killer Cells in Response to Hcv-Infected Hepatoma Cells Is Dependent on Accessory Cells. J Hepatol (2013) 59(3):442–9. doi: 10.1016/j.jhep.2013.04.022 PMC3760030 23665181

[57] Mantovani S Oliviero B Varchetta S Mele D Mondelli MU . Natural Killer Cell Responses in Hepatocellular Carcinoma: Implications for Novel Immunotherapeutic Approaches. Cancers (2020) 12(4):926. doi: 10.3390/cancers12040926 PMC7226319 32283827

[58] Zhang S Liu W Hu B Wang P Lv X Chen S . Prognostic Significance of Tumor-Infiltrating Natural Killer Cells in Solid Tumors: A Systematic Review and Meta-Analysis. Front Immunol (2020) 11:1242. doi: 10.3389/fimmu.2020.01242 32714321 PMC7343909

[59] Zhang QF Yin WW Xia Y Yi YY He QF Wang X . Liver-Infiltrating Cd11b(-)Cd27(-) Nk Subsets Account for Nk-Cell Dysfunction in Patients With Hepatocellular Carcinoma and Are Associated With Tumor Progression. Cell Mol Immunol (2017) 14(10):819–29. doi: 10.1038/cmi.2016.28 PMC5649104 27321064

[60] Nielsen CM White MJ Goodier MR Riley EM . Functional Significance of Cd57 Expression on Human Nk Cells and Relevance to Disease. Front Immunol (2013) 4:422. doi: 10.3389/fimmu.2013.00422 24367364 PMC3856678

[61] Hu G Wang S . Prognostic Role of Tumor-Infiltrating Cd57-Positive Lymphocytes in Solid Tumors: A Meta-Analysis. Oncotarget (2018) 9(8):8111–9. doi: 10.18632/oncotarget.23621 PMC5814286 29487719

[62] Roma S Carpen L Raveane A Bertolini F . The Dual Role of Innate Lymphoid and Natural Killer Cells in Cancer. From Phenotype to Single-Cell Transcriptomics, Functions and Clinical Uses. Cancers (2021) 13(20):5042. doi: 10.3390/cancers13205042 34680190 PMC8533946

[63] Semeraro M Rusakiewicz S Minard-Colin V Delahaye NF Enot D Vély F . Clinical Impact of the Nkp30/B7-H6 Axis in High-Risk Neuroblastoma Patients. Sci Trans Med (2015) 7(283):283ra55. doi: 10.1126/scitranslmed.aaa2327 25877893

[64] Reiners KS Topolar D Henke A Simhadri VR Kessler J Sauer M . Soluble Ligands for Nk Cell Receptors Promote Evasion of Chronic Lymphocytic Leukemia Cells From Nk Cell Anti-Tumor Activity. Blood (2013) 121(18):3658–65. doi: 10.1182/blood-2013-01-476606 PMC3643764 23509156

[65] Schlecker E Fiegler N Arnold A Altevogt P Rose-John S Moldenhauer G . Metalloprotease-Mediated Tumor Cell Shedding of B7-H6, the Ligand of the Natural Killer Cell-Activating Receptor Nkp30. Cancer Res (2014) 74(13):3429–40. doi: 10.1158/0008-5472.Can-13-3017 24780758

[66] Luo Q Luo W Zhu Q Huang H Peng H Liu R . Tumor-Derived Soluble Mica Obstructs the Nkg2d Pathway to Restrain Nk Cytotoxicity. Aging Dis (2020) 11(1):118–28. doi: 10.14336/ad.2019.1017 PMC6961768 32010486

[67] van Montfoort N Borst L Korrer MJ Sluijter M Marijt KA Santegoets SJ . Nkg2a Blockade Potentiates Cd8 T Cell Immunity Induced by Cancer Vaccines. Cell (2018) 175(7):1744–55.e15. doi: 10.1016/j.cell.2018.10.028 30503208 PMC6354585

[68] Borst L van der Burg SH van Hall T . The Nkg2a-Hla-E Axis as a Novel Checkpoint in the Tumor Microenvironment. Clin Cancer Res (2020) 26(21):5549–56. doi: 10.1158/1078-0432.Ccr-19-2095 32409305

[69] Ruggeri L Urbani E André P Mancusi A Tosti A Topini F . Effects of Anti-Nkg2a Antibody Administration on Leukemia and Normal Hematopoietic Cells. Haematologica (2016) 101(5):626–33. doi: 10.3324/haematol.2015.135301 PMC5004363 26721894

[70] Jacquelot N Seillet C Souza-Fonseca-Guimaraes F Sacher AG Belz GT Ohashi PS . Natural Killer Cells and Type 1 Innate Lymphoid Cells in Hepatocellular Carcinoma: Current Knowledge and Future Perspectives. Int J Mol Sci (2021) 22(16):9044. doi: 10.3390/ijms22169044 34445750 PMC8396475

