# Supplementary material for: The Prognostic Value of Natural Killer Cells and Their Receptors/Ligands in Hepatocellular Carcinoma: A Systematic Review and Meta-Analysis
Source: Front Immunol. 2022 Apr 7;13:872353. doi: 10.3389/fimmu.2022.872353 (PMC9021421; doi:10.3389/fimmu.2022.872353)
Supplement: Supplementary file 1 [file DataSheet_1.pdf]

# Supplemental Online Content

Inventory of Supplementary Information

1. eMethods: Search Strategy…………………………………..…..Page 2-4

2. Supporting Table……………………..……………………………Page 5

3. Supporting Figures……………………...………………………...Page 6-10

4. Supporting PRISMA Checklist………………………………..…...Page 11-15

**eMethods: Search Strategy**

**PubMed (396)** (("Carcinoma, Hepatocellular"[Mesh] OR hepatocellular carcinoma OR hepatomas OR liver carcinoma OR liver cell carcinoma OR HCC OR liver cancer) AND (((("Killer Cells, Natural"[Mesh]) OR (NK Cells[Title/Abstract] OR Cell, NK[Title/Abstract] OR Cells, NK[Title/Abstract] OR NK Cell[Title/Abstract] OR Natural Killer Cells[Title/Abstract] OR Cell, Natural Killer[Title/Abstract] OR Cells, Natural Killer[Title/Abstract] OR Killer Cell, Natural[Title/Abstract] OR Natural Killer Cell[Title/Abstract])) OR (("Receptors, Natural Killer Cell"[Mesh]) OR (Natural Killer Cell Receptor[Title/Abstract] OR NK Cell Receptors[Title/Abstract] OR Cell Receptors, NK[Title/Abstract] OR Receptors, NK Cell[Title/Abstract] OR NK Cell Receptor[Title/Abstract] OR Cell Receptor, NK[Title/Abstract] OR Receptor, NK Cell[Title/Abstract] OR Natural Killer Cell Receptors[Title/Abstract] OR Natural Killer Cell Activating Receptors[Title/Abstract] OR Natural Killer Cell Activating Receptor[Title/Abstract] OR Natural Killer Cell Inhibitory Receptors[Title/Abstract] OR Natural Killer Cell Inhibitory Receptor[Title/Abstract]))) OR (CD16[Title/Abstract] OR CD56[Title/Abstract] OR CD57[Title/Abstract]))) AND (survival[Title/Abstract] OR prognosis[Title/Abstract])

**Embase (549)**

#1 'Carcinoma, Hepatocellular'/exp OR 'Carcinoma, Hepatocellular' OR 'hepatocellular carcinoma'/exp OR 'hepatocellular carcinoma' OR hepatomas* OR 'liver carcinoma'/exp OR 'liver carcinoma' OR 'liver cell carcinoma'/exp O 'liver cell carcinoma' OR HCC OR 'liver cancer' (214,178)

#2 'killer cells, natural'/exp OR 'killer cells, natural' OR 'NK Cells':ab,ti OR 'Cell, NK':ab,ti OR 'Cells, NK':ab,ti OR 'NK Cell':ab,ti OR 'Natural Killer Cells':ab,ti OR 'Cell, Natural Killer':ab,ti OR 'Cells, Natural Killer':ab,ti OR 'Killer Cell, Natural':ab,ti OR 'Natural Killer Cell':ab,ti (101,851)

#3 'Receptors, Natural Killer Cell'/exp OR 'Natural Killer Cell Receptor':ab,ti OR 'NK Cell Receptors':ab,ti OR 'Cell Receptors, NK':ab,ti OR 'Receptors, NK Cell':ab,ti OR 'NK Cell Receptor':ab,ti OR 'Cell Receptor, NK':ab,ti OR 'Receptor, NK Cell':ab,ti OR 'Natural Killer Cell Receptors':ab,ti OR 'Natural Killer Cell Activating Receptors':ab,ti OR 'Natural Killer Cell Activating Receptor':ab,ti OR 'Natural Killer Cell Inhibitory Receptors':ab,ti OR 'Natural Killer Cell Inhibitory Receptor':ab,ti (3,489)

#4 'CD16':ab,ti OR 'CD56':ab,ti OR 'CD57':ab,ti (30,418)

#5 survival :ab,ti OR prognosis :ab,ti (1,976,694)

#6 #2 OR #3 OR #4 (120,433)

#7 #1 AND #6 AND #5 (549)

**Web of science (841)**

1 TS=(carcinoma, hepatocellular) OR TS=(hepatocellular carcinoma) OR TS=(hepatomas*) OR TS=(liver carcinoma) OR TS=(liver cell carcinoma) OR TS=(HCC) OR TS=(liver cancer) (444,669)

2 TS=(Killer Cells, Natural) OR TI=("NK Cells" OR "Cell, NK" OR "Cells, NK" OR "NK Cell" OR "Natural Killer Cells" OR "Cell, Natural Killer" OR "Cells, Natural Killer" OR "Killer Cell, Natural" OR "Natural Killer Cell") OR AB=("NK Cells" OR "Cell, NK" OR "Cells, NK" OR "NK Cell" OR "Natural Killer Cells" OR (121,807)

3 TS=(Receptors, Natural Killer Cell) OR TI=("Natural Killer Cell Receptor" OR "NK Cell Receptors" OR "Cell Receptors, NK" OR "Receptors, NK Cell" OR "NK Cell Receptor" OR "Cell Receptor, NK" OR "Receptor, NK Cell" OR "Natural Killer Cell Receptors" OR "Natural Killer Cell Activating Receptors" OR "Natural Killer Cell Activating Receptor" OR "Natural Killer Cell Inhibitory Receptors" OR "Natural Killer Cell Inhibitory Receptor") OR AB=("Natural Killer Cell Receptor" OR "NK Cell Receptors" OR "Cell Receptors, NK" OR "Receptors, NK Cell" OR "NK Cell Receptor" OR "Cell Receptor, NK" OR "Receptor, NK Cell" OR "Natural Killer Cell Receptors" OR "Natural Killer Cell Activating Receptors" OR "Natural Killer Cell Activating Receptor" OR "Natural Killer Cell Inhibitory Receptors" OR "Natural Killer Cell Inhibitory Receptor") (42,298)

4 TI=(CD16 OR CD56 OR CD57) OR AB=(CD16 OR CD56 OR CD57) (24,149)

5 TI=(survival OR prognosis) OR AB=(survival OR prognosis) (1,933,147)

6 2 OR 3 OR 4 (134,895)

7 1 AND 6 AND 5 (841)

**Cochrane Library literature databases (44)**

1 MeSH descriptor: [Carcinoma, Hepatocellular] explode all trees (1,866)

2 (hepatocellular carcinoma OR hepatomas OR liver carcinoma OR liver cell carcinoma OR HCC OR liver cancer):ti,ab,kw (7941)

3 MeSH descriptor:[Killer Cells, Natural] explode all trees (791)

4 (NK Cells OR Cell, NK OR Cells, NK OR NK Cell OR Natural killer Cells OR Cell, Natural Killer OR Cells, Natural Killer OR Killer Cell Natural OR Natural killer (3,138)

5 MeSH descriptor: [Receptors, Natural Killer Cell] explode all trees (29)

6 (Natural Killer Cell Receptor OR NK Cell Receptors OR Cell Receptors, NK OR Receptors, NK Cell OR NK Cell Receptor OR Cell Receptor, NK OR Receptor, NK Cell OR Natural killer Cell Receptors OR Natural Killer Cell Activating Receptors OR Natural killer Cell Activating Receptor OR Natural Killer Cell inhibitory Receptors OR Natural Killer Cell inhibitory Receptor):ti,ab,kw (488)

7 (survival OR prognosis):ti,ab,kw (135,349)

8 (CD16 OR CD56 OR CD57):ti,ab,kw (954)

9 1 OR 2 (7,941)

10 3 OR 4 (3,172)

11 5 OR 6 (493)

12 8 OR 10 OR 11 (3,660)

13 9 AND 12 AND 7 (44)

supplementary Table 1 Quality assessment of studies by checklist (based on Newcastle-Ottawa Scale)

| Study | Selection | | | | Comparability | Outcome | | | Score |
|---|---|---|---|---|---|---|---|---|---|
| | I | II | III | IV | V | VI | VII | VIII | |
| Zhuang et al.2019 | ☆ | ★ | ★ | ★ | ★ | ★ | ★ | ★ | 7 |
| Hu et al.2021 | ★ | ★ | ★ | ★ | ☆☆ | ★ | ★ | ★ | 7 |
| Lin et al.2013 | ★ | ★ | ★ | ★ | ★ | ★ | ★ | ★ | 8 |
| Wu et al.2013 | ★ | ★ | ★ | ★ | ★★ | ★ | ★ | ★ | 9 |
| Tao et al.2020 | ★ | ★ | ★ | ★ | ★★ | ★ | ★ | ★ | 9 |
| Chew et al.2012 | ☆ | ★ | ★ | ★ | ★ | ☆ | ★ | ★ | 6 |
| Zhao et al.2014 | ★ | ★ | ★ | ★ | ★★ | ★ | ★ | ★ | 9 |
| Gao et al.2012 | ★ | ★ | ★ | ★ | ★ | ★ | ★ | ★ | 8 |
| Cariani et al.2016 | ☆ | ★ | ★ | ★ | ★ | ★ | ★ | ★ | 7 |
| Pan et al.2014 | ★ | ★ | ★ | ★ | ★★ | ★ | ★ | ★ | 9 |
| Liu et al.2021 | ★ | ★ | ★ | ★ | ★★ | ☆ | ☆ | ☆ | 6 |
| Pan et al.2020 | ☆ | ★ | ★ | ★ | ★★ | ☆ | ★ | ★ | 7 |
| Che et al.2014 | ☆ | ★ | ★ | ★ | ★★ | ★ | ☆ | ☆ | 6 |

**Abbreviations**: I, Representativeness of the exposed cohort; II, Selection of the nonexposed cohort; III, Ascertainment of exposure; IV, Demonstration that outcome of interest was not present at start of study; V, Comparability of cases and controls on the basis of the design or analysis; VI, Assessment of outcome; VII, Follow-up long enough for outcomes to occur; VIII, Adequacy of follow up of cohorts;

★, Asterisk means that the study is satisfied the item; ☆, asterisk means the opposite situation; A score > 6 was defined as high quality, and ≤ 6 was defined as low quality.

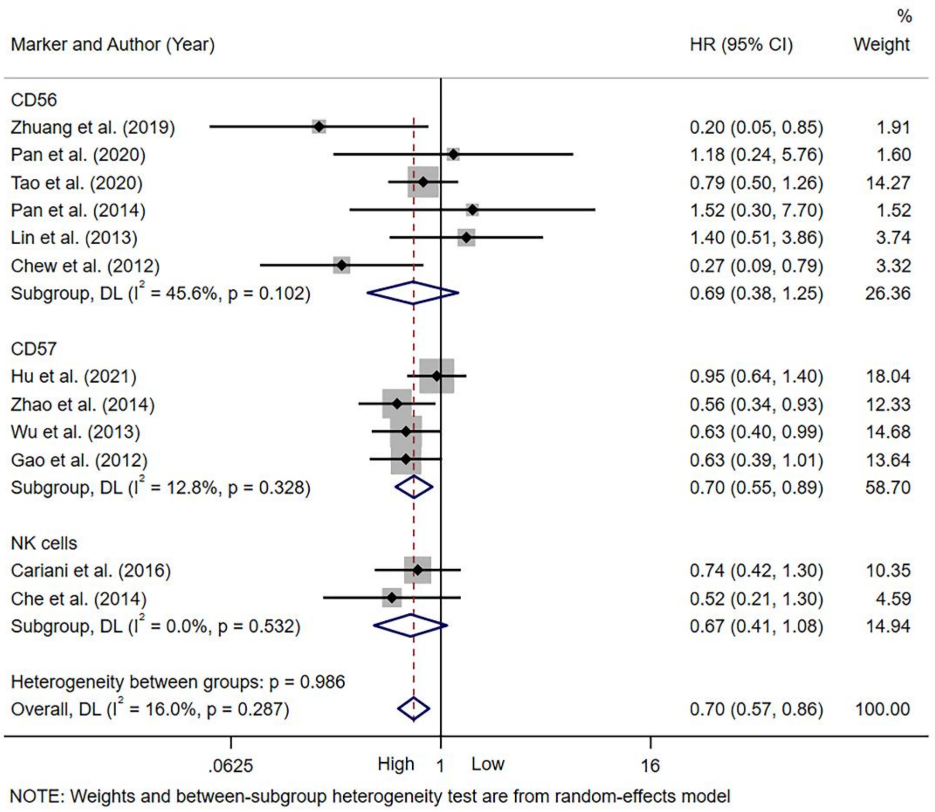


supplementary Figure 1: Forest plot of NK cells marker and OS in hepatocellular carcinoma. CI, Confidence interval; HR, Hazard ratio; OS, Overall survival; NK, Natural killer

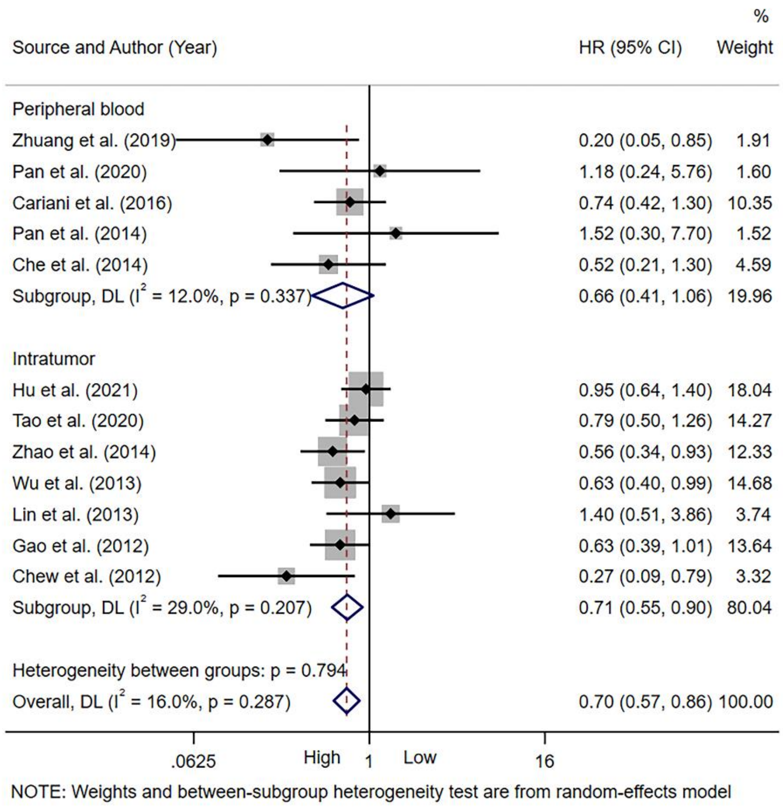


supplementary Figure 2: Forest plot of NK cells source and OS in hepatocellular carcinoma. CI, Confidence interval; HR, Hazard ratio; OS, Overall survival; NK, Natural killer

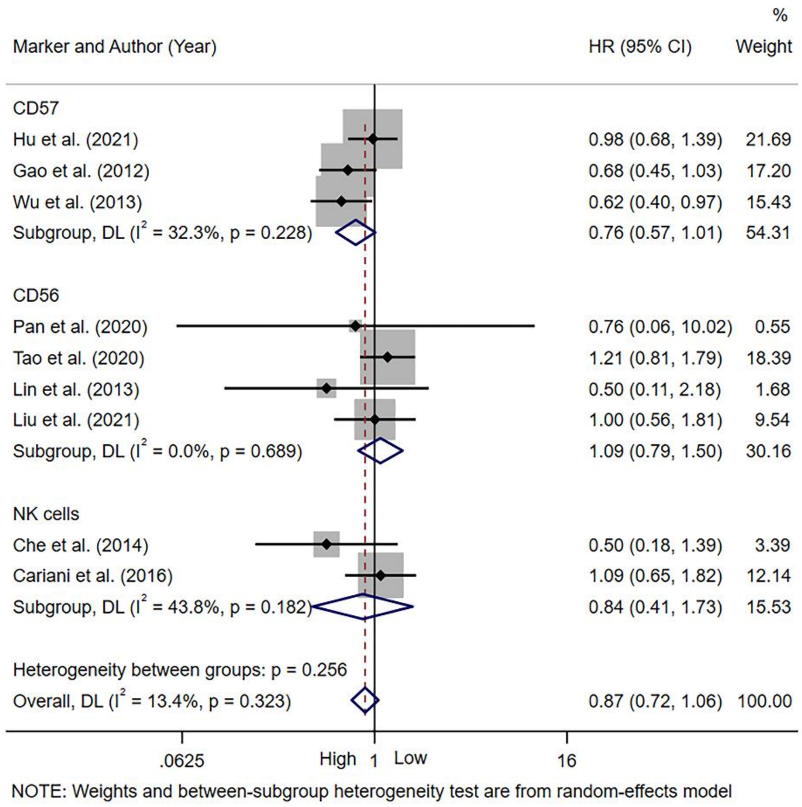


supplementary Figure 3: Forest plot of NK cells marker and DFS/RFS/TTR/PFS in hepatocellular carcinoma. CI, Confidence interval; HR, Hazard ratio; NK, Natural killer; DFS, Disease-free survival; RFS, Recurrence-free survival; TTR, Time-to recurrence; PFS, Progression-free survival

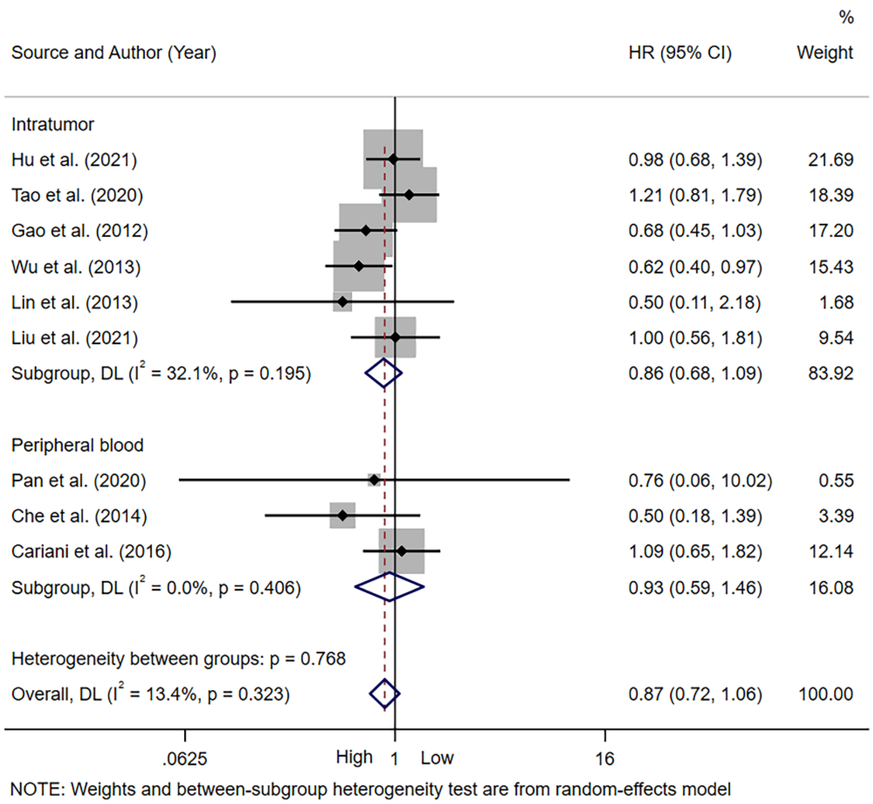


supplementary Figure 4: Forest plot of NK cells source and DFS/RFS/TTR/PFS in hepatocellular carcinoma. CI, Confidence interval; HR, Hazard ratio; NK, Natural killer; DFS, Disease-free survival; RFS, Recurrence-free survival; TTR, Time-to recurrence; PFS, Progression-free survival

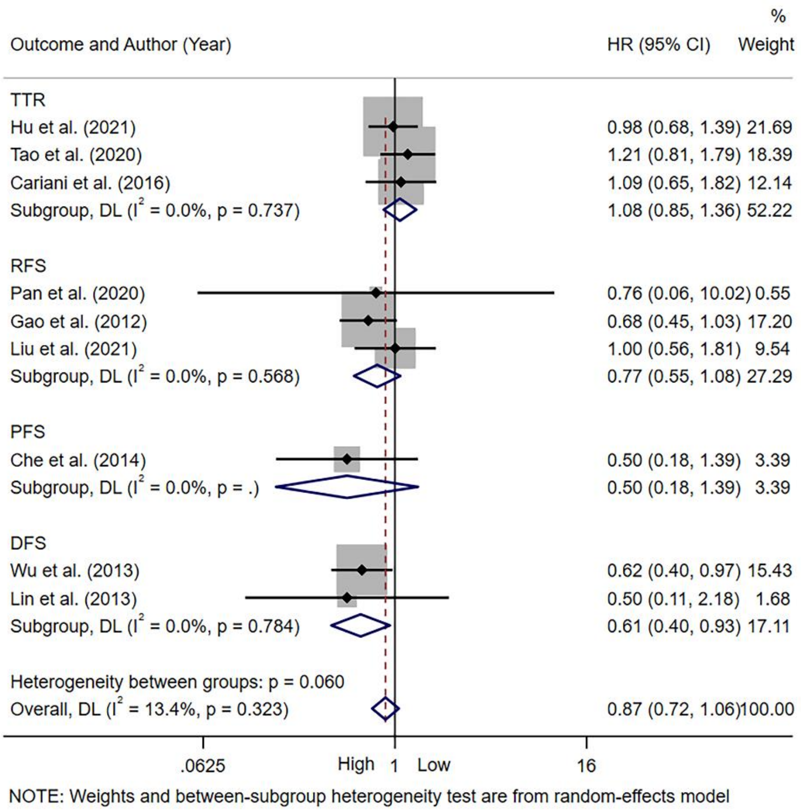


supplementary Figure 5: Forest plot of NK cells outcome in hepatocellular carcinoma. CI, Confidence interval; HR, Hazard ratio; NK, Natural killer; DFS, Disease-free survival; RFS, Recurrence-free survival; TTR, Time-to recurrence; PFS, Progression-free survival

Supplementary PRISMA Checklist

| Section/topic | # | Checklist item | Reported on page # |
|---|---|---|---|
| **TITLE** | | | |
| Title | 1 | Identify the report as a systematic review and meta-analysis. | 1 |
| **ABSTRACT** | | | |
| Structured summary | 2 | Provide a structured summary including, as applicable: background; objectives; data sources; study eligibility criteria, participants, and interventions; study appraisal and synthesis methods; results; limitations; conclusions and implications of key findings; systematic review registration number. | 3-4 |
| **INTRODUCTION** | | | |
| Rationale | 3 | Describe the rationale for the review in the context of what is already known. | 4-6 |
| Objectives | 4 | Provide an explicit statement of questions being addressed with reference to participants, interventions, comparisons, outcomes, and study design (PICOS). | 6 |
| **METHODS** | | | |
| Protocol and registration | 5 | Indicate if a review protocol exists, if and where it can be accessed (e.g., Web address), and, if available, provide registration information including registration number. | N/A |
| Eligibility criteria | 6 | Specify study characteristics (e.g., PICOS, length of follow-up) and report characteristics (e.g., years considered, language, publication status) used as | 7 |

| | | | |
|---|---|---|---|
| | | criteria for eligibility, giving rationale. | |
| Information sources | 7 | Describe all information sources (e.g., databases with dates of coverage, contact with study authors to identify additional studies) in the search and date last searched. | 6-7 |
| Search | 8 | Present full electronic search strategy for at least one database, including any limits used, such that it could be repeated. | 6-7 |
| Study selection | 9 | State the process for selecting studies (i.e., screening, eligibility, included in systematic review, and, if applicable, included in the meta-analysis). | 7-8 |
| Data collection process | 10 | Describe method of data extraction from reports (e.g., piloted forms, independently, in duplicate) and any processes for obtaining and confirming data from investigators. | 7 |
| Data items | 11 | List and define all variables for which data were sought (e.g., PICOS, funding sources) and any assumptions and simplifications made. | 8 |
| Risk of bias in individual studies | 12 | Describe methods used for assessing risk of bias of individual studies (including specification of whether this was done at the study or outcome level), and how this information is to be used in any data synthesis. | 8 |
| Summary measures | 13 | State the principal summary measures (e.g., risk ratio, difference in means). | 8 |

| | | | |
|---|---|---|---|
| Synthesis of results | 14 | Describe the methods of handling data and combining results of studies, if done, including measures of consistency (e.g., $I^2$) for each meta-analysis. | 8 |



| Section/topic | # | Checklist item | Reported on page # |
|---|---|---|---|
| Risk of bias across studies | 15 | Specify any assessment of risk of bias that may affect the cumulative evidence (e.g., publication bias, selective reporting within studies). | 8 |
| Additional analyses | 16 | Describe methods of additional analyses (e.g., sensitivity or subgroup analyses, meta-regression), if done, indicating which were pre-specified. | 8 |
| **RESULTS** | | | |
| Study selection | 17 | Give numbers of studies screened, assessed for eligibility, and included in the review, with reasons for exclusions at each stage, ideally with a flow diagram. | 9 |
| Study characteristics | 18 | For each study, present characteristics for which data were extracted (e.g., study size, PICOS, follow-up period) and provide the citations. | 9 |
| Risk of bias within studies | 19 | Present data on risk of bias of each study and, if available, any outcome level assessment (see item 12). | 9-10 |
| Results of individual studies | 20 | For all outcomes considered (benefits or harms), present, for each study: (a) simple summary data for each intervention group (b) effect | 10-12 |

| | | | |
|---|---|---|---|
| | | estimates and confidence intervals, ideally with a forest plot. | |
| Synthesis of results | 21 | Present results of each meta-analysis done, including confidence intervals and measures of consistency. | 10-12 |
| Risk of bias across studies | 22 | Present results of any assessment of risk of bias across studies (see Item 15). | 12 |
| Additional analysis | 23 | Give results of additional analyses, if done (e.g., sensitivity or subgroup analyses, meta-regression [see Item 16]). | 10-12 |
| **DISCUSSION** | | | |
| Summary of evidence | 24 | Summarize the main findings including the strength of evidence for each main outcome; consider their relevance to key groups (e.g., healthcare providers, users, and policy makers). | 13-15 |
| Limitations | 25 | Discuss limitations at study and outcome level (e.g., risk of bias), and at review-level (e.g., incomplete retrieval of identified research, reporting bias). | 15-16 |
| Conclusions | 26 | Provide a general interpretation of the results in the context of other evidence, and implications for future research. | 16-17 |
| **FUNDING** | | | |
| Funding | 27 | Describe sources of funding for the systematic review and other support (e.g., supply of data); role of funders for the systematic review. | 1-2 |

*From:* Moher D, Liberati A, Tetzlaff J, Altman DG, The PRISMA Group (2009). Preferred Reporting Items for Systematic Reviews and Meta-Analyses: The PRISMA Statement. PLoS Med 6(7): e1000097.

doi:10.1371/journal.pmed1000097
